# Supplementary material for: Seminoma and Embryonal Carcinoma Footprints Identified by Analysis of Integrated Genome-Wide Epigenetic and Expression Profiles of Germ Cell Cancer Cell Lines
Source: PLoS One. 2014 Jun 2;9(6):e98330. doi: 10.1371/journal.pone.0098330 (PMC4041891; doi:10.1371/journal.pone.0098330)
Supplement: File S1 — ZIP file containing DMRforPairs output for significant regions. Please start from the html files. (ZIP) [file pone.0098330.s008.zip › figures/40616.pdf]

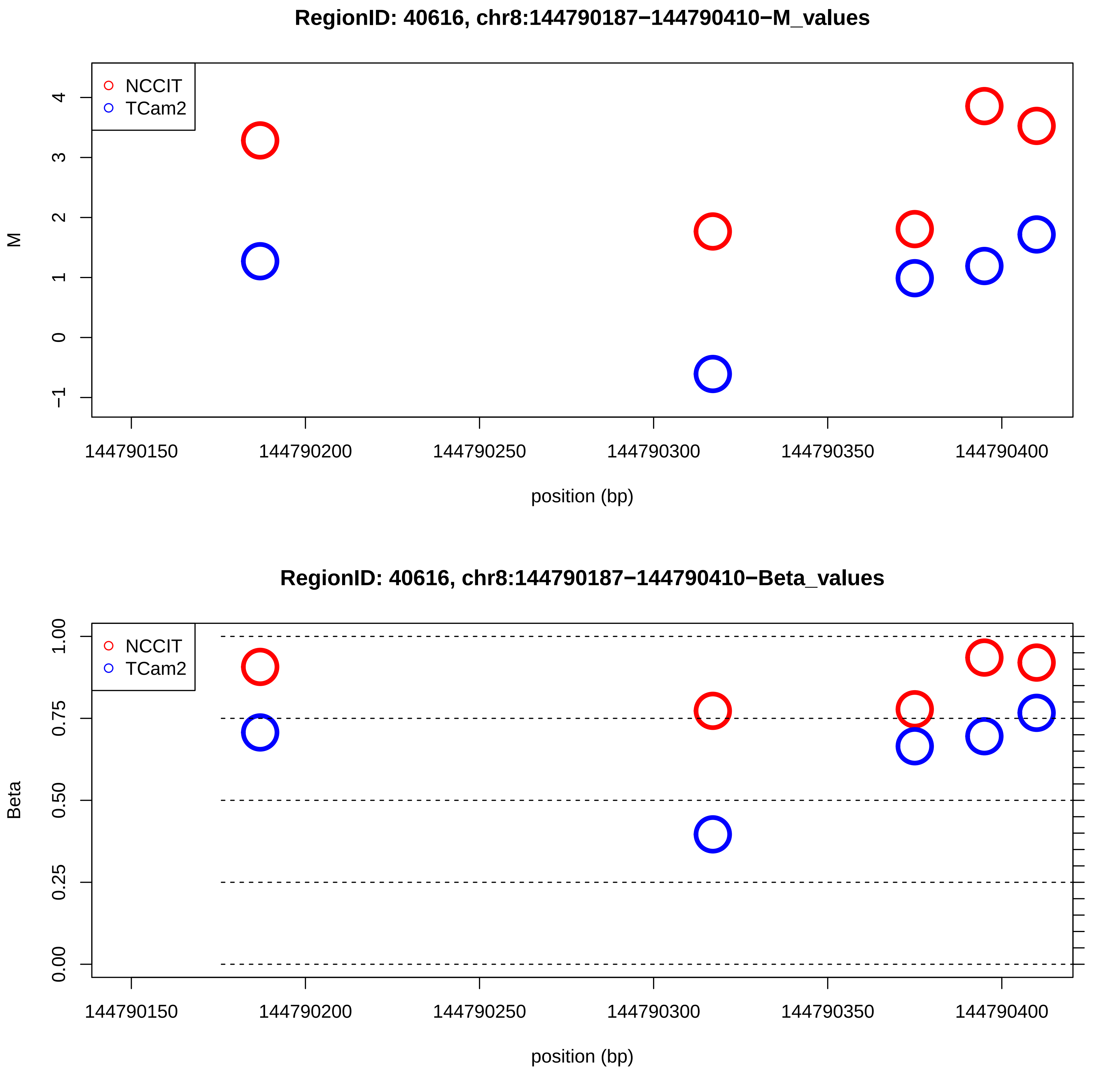

RegionID: 40616, chr8:144790187−144790410−M_values
NCCIT
TCam2
M
4
3
2
1
0
−1
144790150
144790200
144790250
144790300
144790350
144790400
position (bp)
RegionID: 40616, chr8:144790187−144790410−Beta_values
NCCIT
TCam2
Beta
1.00
0.75
0.50
0.25
0.00
144790150
144790200
144790250
144790300
144790350
144790400
position (bp)
